# Diabetes Management in Severe Mental Illness: An Audit at Broadmoor Hospital

**DOI:** 10.1192/j.eurpsy.2026.10969

**Published:** 2026-07-31

**Authors:** S. Olotu, I. Hussein, S. Jyoti

**Affiliations:** Broadmoor Hospital, West London NHS, Berkshire, United Kingdom

## Abstract

**Introduction:**

Individuals with severe mental illness (SMI) face a 13–30 year mortality gap, with type 2 diabetes contributing significantly due to lifestyle, antipsychotic medication use, and limited access to equitable physical healthcare. At Broadmoor Hospital, concerns about diabetes complications and an emergency hyperglycaemic episode prompted this audit. Raising awareness about diabetes education among mental health professionals and patients is a top priority

**Objectives:**

Determine the prevalence of type 2 diabetes and related complications in Broadmoor Hospital

1. Evaluate the education and knowledge of diabetes among the patients and nursing staff
2. Gaps in current care and recommendations in line with the National Institute of Clinical Excellence (NICE) guideline and the Public Health guideline [PH38] Type 2 diabetes: prevention in people at high risk

**Methods:**

Data collection (Oct 2024–Mar 2025) included review of patient health records and HbA1c monitoring Evidence-based surveys were distributed to 106 nursing staff (20.7% response rate) and 21 inpatients with type 2 diabetes Quantitative and qualitative data analysis, incorporating the Theoretical Domains Framework (TDF), was used to interpret the findings (Image 3)

**Results:**

**Staff survey:** 54% of nurses had received diabetes training; only 21% within the past year. Nearly half were unsure of guidelines, with 43% citing lack of training as a barrier. Patient compliance (84.9%) was the most reported challenge, followed by workload pressures and limited resources. Nurses strongly supported structured training and better patient education resources (Image 1&2)

**Patient survey:** Most patients felt knowledgeable and motivated to manage diabetes, yet 71% reported never being offered a structured education course. Exercise (61%) and diet adherence (22%) were the greatest challenges. Mental health significantly influenced self-management—75% reported poor mental health worsened diabetes control. While most patients were confident and optimistic, gaps between confidence and routine adherence persisted (Image 1)

Significant correlation was observed between patient confidence and optimism (r ≈ 0.68, p < 0.01). Lack of structured education was statistically linked to unmet educational needs (p < 0.05)

**Image 1:**
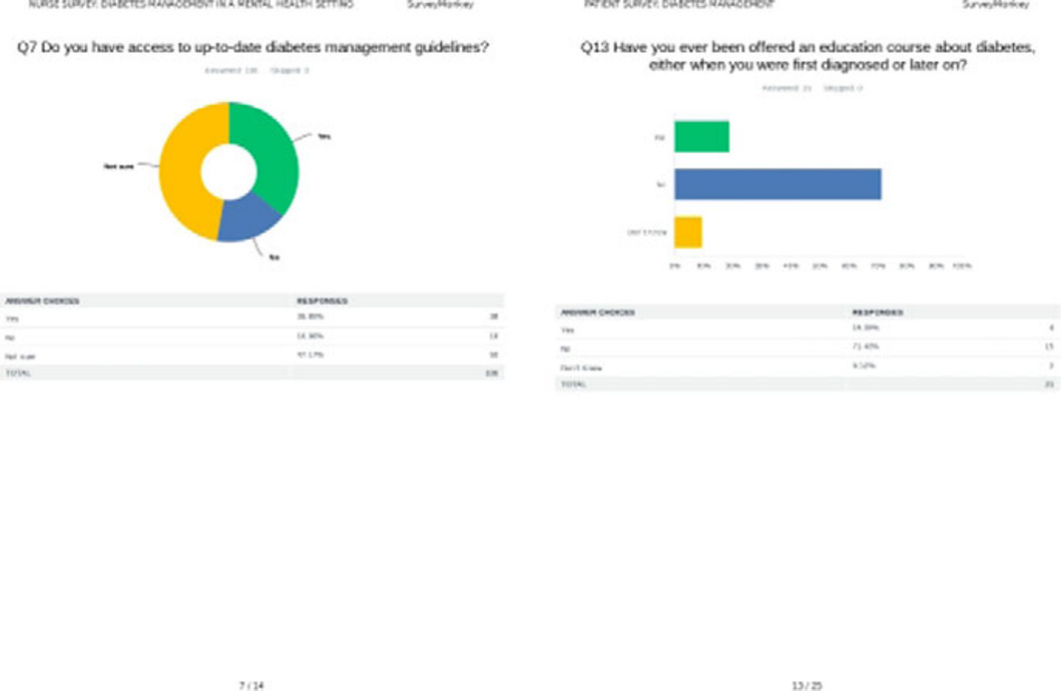
Image 1: Long description.

**Image 2:**
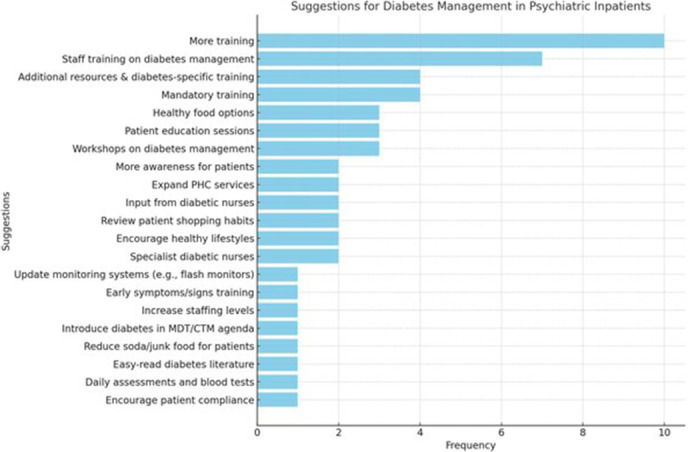
Image 2: Long description.

**Image 3:**
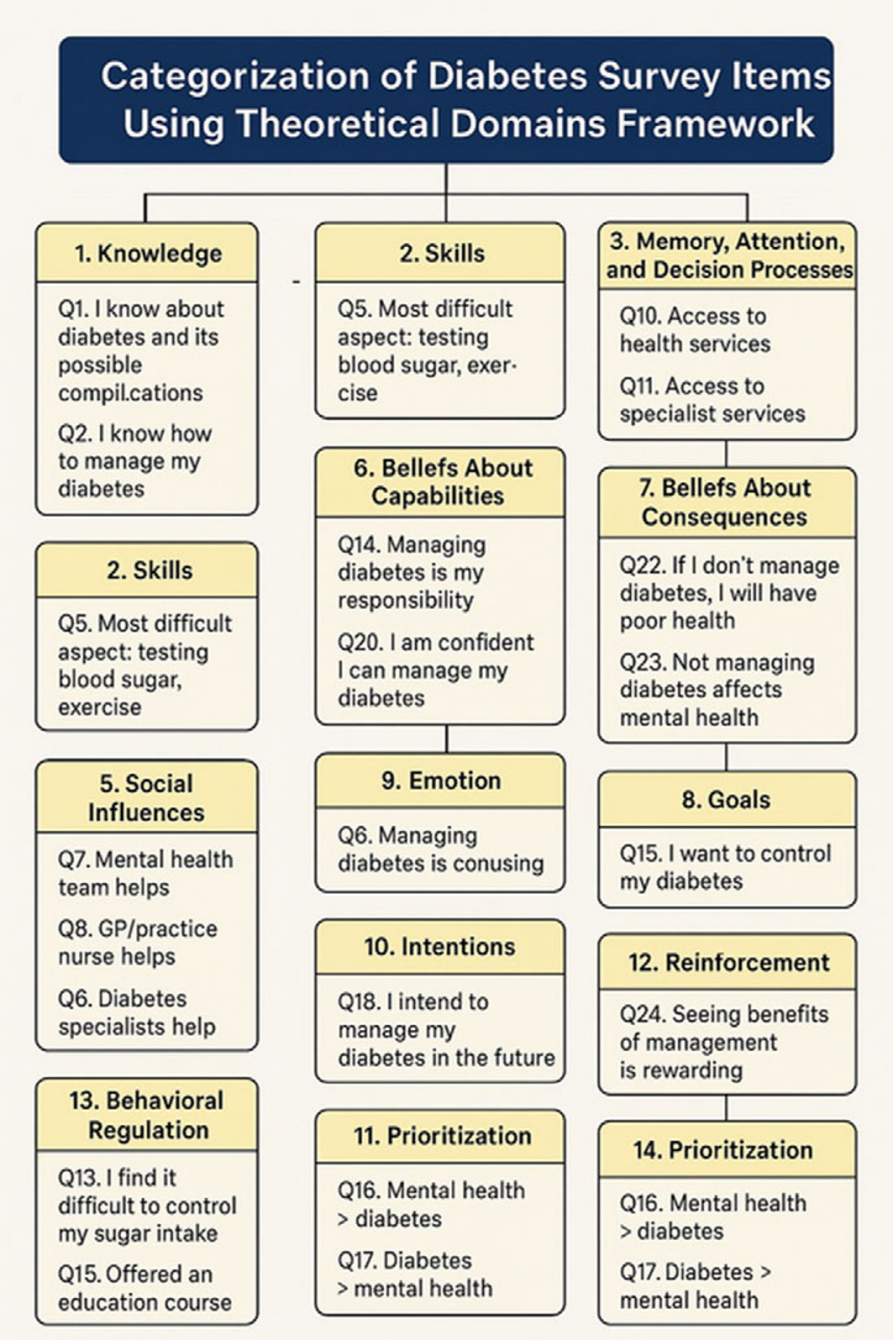
Image 3: Long description.

**Conclusions:**

This audit highlights major gaps in structured diabetes education for both staff and patients in a secure forensic setting. Despite good awareness and motivation, barriers such as limited training, patient compliance, mental health challenges, and lack of structured support hinder effective management

**Recommendations:**

- Implement mandatory diabetes training and refresher courses for nurses
- Develop structured patient education programmes (e.g., DESMOND model)
- Enhance collaboration within the multidisciplinary team

**Impact:**

Embedding the recommended approaches could improve diabetes outcomes, reduce complications, and address health inequalities for individuals with SMI in secure settings

**Disclosure of Interest:**

None Declared

## Long descriptions

### Image 1: Long description

The figure contains two panels from SurveyMonkey reports.

Left Panel: Nurse Survey: Diabetes Management in a Mental Health Setting.

Question 7 asks, Do you have access to up-to-date diabetes management guidelines?

Answered: 106. Skipped: 0.

A donut chart shows three segments.

* Yes (green): 35.85 percent, 38 responses.

* No (blue): 16.98 percent, 18 responses.

* Not sure (yellow): 47.17 percent, 50 responses.

A table below confirms these values with a total of 106.

Right Panel: Patient Survey: Diabetes Management.

Question 13 asks, Have you ever been offered an education course about diabetes, either when you were first diagnosed or later on?

Answered: 21. Skipped: 0.

A horizontal bar chart shows three bars.

* Yes (green): 19.05 percent, 4 responses.

* No (blue): 71.43 percent, 15 responses.

* Don't know (yellow): 9.52 percent, 2 responses.

A table below confirms these values with a total of 21. The X axis of the bar chart ranges from 0 percent to 100 percent in 10 percent increments.

Navigate back to Image 1:.

### Image 2: Long description

The horizontal bar chart plots Suggestions on the y-axis against Frequency on the x-axis, which ranges from 0 to 10. The data is presented in descending order of frequency:

* More training: 10

* Staff training on diabetes management: 7

* Additional resources and diabetes-specific training: 4

* Mandatory training: 4

* Healthy food options: 3

* Patient education sessions: 3

* Workshops on diabetes management: 3

* More awareness for patients: 2

* Expand P H C services: 2

* Input from diabetic nurses: 2

* Review patient shopping habits: 2

* Encourage healthy lifestyles: 2

* Specialist diabetic nurses: 2

* Update monitoring systems (e.g., flash monitors): 1

* Early symptoms/signs training: 1

* Increase staffing levels: 1

* Introduce diabetes in M D T / C T M agenda: 1

* Reduce soda/junk food for patients: 1

* Easy-read diabetes literature: 1

* Daily assessments and blood tests: 1

* Encourage patient compliance: 1

Navigate back to Image 2:.

### Image 3: Long description

A flowchart titled Categorization of Diabetes Survey Items Using Theoretical Domains Framework. The diagram is organized into three vertical columns branching from the main title.

Left Column:

* 1. Knowledge: contains Q 1. I know about diabetes and its possible complications and Q 2. I know how to manage my diabetes.

* 2. Skills: contains Q 5. Most difficult aspect: testing blood sugar, exercise.

* 5. Social Influences: contains Q 7. Mental health team helps, Q 8. G P / practice nurse helps, and Q 6. Diabetes specialists help.

* 13. Behavioral Regulation: contains Q 13. I find it difficult to control my sugar intake and Q 15. Offered an education course.

Middle Column:

* 2. Skills: contains Q 5. Most difficult aspect: testing blood sugar, exercise.

* 6. Beliefs About Capabilities: contains Q 14. Managing diabetes is my responsibility and Q 20. I am confident I can manage my diabetes.

* 9. Emotion: contains Q 6. Managing diabetes is confusing.

* 10. Intentions: contains Q 18. I intend to manage my diabetes in the future.

* 11. Prioritization: contains Q 16. Mental health > diabetes and Q 17. Diabetes > mental health.

Right Column:

* 3. Memory, Attention, and Decision Processes: contains Q 10. Access to health services and Q 11. Access to specialist services.

* 7. Beliefs About Consequences: contains Q 22. If I don't manage diabetes, I will have poor health and Q 23. Not managing diabetes affects mental health.

* 8. Goals: contains Q 15. I want to control my diabetes.

* 12. Reinforcement: contains Q 24. Seeing benefits of management is rewarding.

* 14. Prioritization: contains Q 16. Mental health > diabetes and Q 17. Diabetes > mental health.

Navigate back to Image 3:.

